# Ultrafine PVDF Nanofibers for Filtration of Air-Borne Particulate Matters: A Comprehensive Review

**DOI:** 10.3390/polym13111864

**Published:** 2021-06-03

**Authors:** Ayishe Sanyal, Sumit Sinha-Ray

**Affiliations:** 1 School of Engineering, Indian Institute of Technology Mandi, Mandi 175005, HP, India; d20016@students.iitmandi.ac.in; 2 Department of Mechanical and Industrial Engineering, University of Illinois at Chicago, Chicago, IL 60607-7022, USA

**Keywords:** PVDF, ultrafine fibers, particulate matter, electret, slip flow

## Abstract

The ongoing global pandemic has bestowed high priority uponthe separation of air-borne particulate matters (PMs), aerosols, etc. using nonwoven fibrous materials, especially for face masks as a means of personal protection. Although spunbond or meltblown nonwoven materials are amongst the forerunners for polymer microfiber-based face mask or air filter development in mass scale, relatively new process of nonwoven manufacturing such as electrospinning is gaining a lot of momentum amongst the filter membrane manufacturers for its scalability of nanofiber-based filter membrane fabrication. There are several nanofiber-based face masks developing industries, which claim a very high efficiency in filtration of particulate matters (PM_0.1–10_) as well as other aerosols for their products. Polyvinylidene fluoride (PVDF), which is commonly known for its use of tactile sensors and energy harvesters, due to its piezoelectric property, is slowly gaining popularity among researchers and developers as an air filter material. Electrospun PVDF nanofibers can be as fine as 50 nm in mass scale, which allows the membrane to have large surface area compared to its volume, enhancing nanofiber–PM interaction. At the same time, the breathability index can be improved through these PVDF nanofiber membranes due to their architectural uniqueness that promotes slip flow around the fibers. The conductive nature of PVDF makes it advantageous as a promising electret filter allowing better capturing of ultrafine particles. This review aims to provide a comprehensive overview of such PVDF nanofiber-based filter membranes and their roles in air filtration, especially its application in filtrate of air-borne PMs.

## 1. Introduction

In the era of industrialization, automobile growth and over dependence of fossil fuel has led to a severe scenario of air pollution, one of the most severe threats to human race. According to a data published by World Health Organization (WHO), about 90% of people breathe polluted air across the globe and air pollution alone is the sole cause of more than seven million deaths every year, as per the same data [1]. From outdoor air pollution to indoor smoke, the situation has become grimmer every year leading to increased cases of respiratory damages. The extent of air pollution is generally quantified using a parameter called air quality index (AQI), based on primarily five components—sulfur oxides (SO_x_), nitrogen oxides (NO_x_), carbon monoxide (CO), particulate matters (PM), and ozone (O_3_), along with volatile organic compounds (VOC) and can be measured as stated in Equation (1) [2].

(1) $$\mathrm{AQI}=\frac{\left(I_{\mathrm{Hi}}-I_{\mathrm{Lo}}\right)}{\left(BP_{\mathrm{Hi}}-BP_{\mathrm{Lo}}\right)}\left(C_{P}-BP_{\mathrm{Lo}}\right)+I_{\mathrm{Lo}},$$

where P stands for the pollutant, C_P_ is the concentration of pollutant, BP_Hi_ and BP_Lo_ are breakpoint concentrations greater and smaller compared to C_P_, respectively, I_Hi_ and I_Lo_ are AQI values corresponding to BP_Hi_ and BP_Lo_, respectively. The AQI is broadly divided into five categories: namely, good (0–50), satisfactory (50–100), moderate (101–200), poor (201–300), very poor (301–400), and severe (401–500) [2].

**Figure 1:**
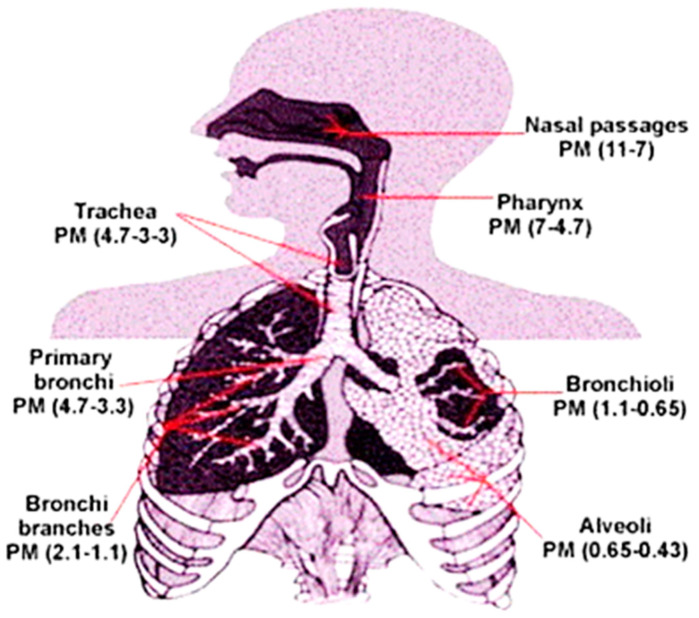
Deposition of PMs of different sizes through a human respiratory tract. Reproduced with permission from Ref. [3]. Copyright Elsevier, 2015.

Out of all the pollutants, the particulate matters lead to more pronounced and sustaining danger to human health, as PMs are often a mixture of various solids and liquids, with various chemical composition, dependent upon the area where they are generated [3]. The crop stubbles, vehicular emissions, and industrial exhausts containing NO_x_, SO_x_, aromatic hydrocarbons, metals, etc. are the constituents of PMs, which can be broadly classified into primary type, i.e., air-borne particulates arising from anthropogenic sources, and secondary type, i.e., gaseous precursor/pollutants [4]. PMs are mainly divided into three categories based on their sizes—PM_10_ (≤10 μm), PM_2.5_ (≤2.5 μm), and ≤PM_1.0_ (1 μm), especially PM_0.1_ (≈0.1 μm), are, respectively, called thoracic, fine, and ultrafine particles [5]. Although the AQI values are routinely reported in terms of PM_10_, sensitive measurements can include PM_2.5_ measurements. As an example: On December 30, 2019, the AQI values across Delhi, India, varied between 430 and 490 as an average, rendering the atmosphere severely polluted [6], where the primary pollutant was PM_2.5_. As per the WHO guidelines, the 24 h average limit of PM_10_ and PM_2.5_ should be <50 and 25 μg/m^3^, respectively. The larger particulate matters tend to settle across the respiratory tracts, whereas the ultrafine ones tend to penetrate the alveoli [7]. A schematic is shown in Figure 1 to demonstrate the levels of PM deposition across various depth of human respiratory tract. The alarming rise of PMs has been found to cause respiratory troubles, acute coronary disorder (ACD), lung inflammation, pulmonary disorders, cancer, and even death amidst children and elderly people [8]. 

**Figure 2:**
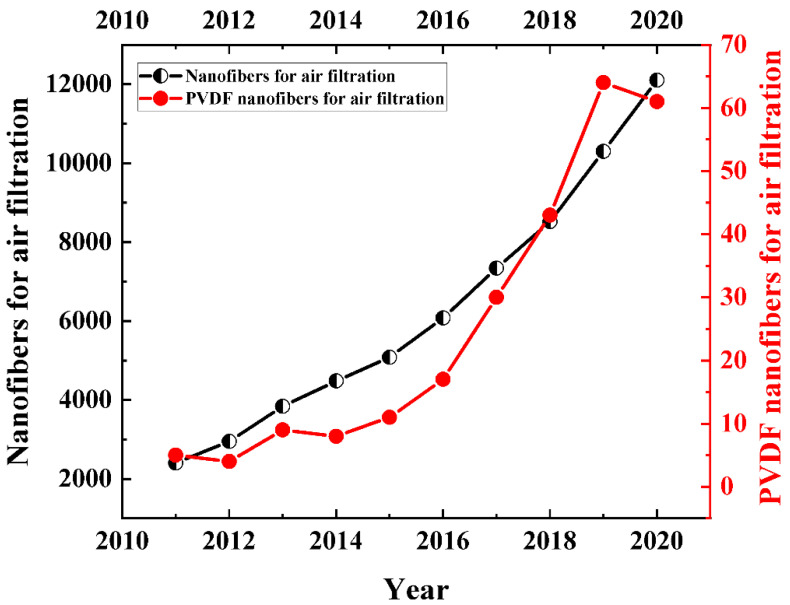
Comparison between annual publication of scientific journals from 2011 to 2020 between ultrafine fibers in general and ultrafine PVDF fibers in air filters. (Obtained from Web of Science search system (3 March 2021) where the keywords were chosen as “ultrafine fibers in air filters” and “ultrafine PVDF fibers in air filters”.)

Although this review focuses upon the removal of PMs, which were so far classified mostly based on the effects of industrialization or urbanization, along with crop burning, etc., the effects of bio-aerosols, originating from coughs, sneezes, or regular breathing, cannot be ruled out, especially in the backdrop of the recent pandemic. The lifetime of aerosols/droplets may not be as long as PM generated from the earlier mentioned sources, but their effects can be lethal, which has been already established without an iota of doubt. These aerosols can easily be modelled similarly as PMs and hence the filtering mechanisms are expected to be similar to other anthropogenically created PMs. While the world is fighting with this pandemic, the growth of nonwoven fiber-based face mask has completely swept the market as the single most important personal protective item. Albeit the boom of N95 or N99 mask market is more prominent now, spunbond or meltblown fiber-based membrane filters were developed and extensively used from a few decades ago. These nonwoven-based face masks or air filters such as new melt-blown fiber-based high efficiency particulate air (HEPA) filters [9] take preventive measure against air pollution. The standard polymers are polypropylene (PP), polyester (PET), nylon, etc., which are mostly spun up into 1–5 μm thick fibers via meltblowing. However, for improved filtration efficiency at a low pressure drop, ultrafine fibers have started substituting a few microns thick conventional fibrous respirators in the past decade as evident from Figure 2, for their potential as nanofibrous or nanocomposite filters with the highest efficiency of ~99.97%, meeting the requirements of a HEPA filter [10]. At the same time, there is a simultaneous rise of development of self-polarized electret nanofilters, using piezoelectric polymer such as polyvinylidene fluoride (PVDF) [11], which is otherwise mostly known for its energy harvesting application. 

Although the domain of PMs and their removal using fibrous filter is a vast and interesting field, with several articles available covering the basics of theoretical development to experimental verification of air filtration, no single comprehensive review is available that emphasizes the application of PVDF-based filters only, letting alone the ultrafine nanofiber-based filters. For the sake of brevity and within the boundary of the scope of this review article, the discussion on effect of ultrafine fibers (here and here after, the ultrafine fibers will mostly indicate to ~10–500 nm diameter scale) for PM removal is mostly restricted for PVDF, because of its unique architectural benefits and its capability to be extruded into the desired size range with a right choice of solvents. Even though this review is focusing on a narrow domain of ultrafine fibers or nanofibers of a particular polymer, the emergence of this field is evidenced via Figure 2. Hence, this review will systematically cover the manufacturing techniques to fabricate ultrafine fibers, followed by the mechanisms of PM capturing by ultrafine fibers and various kinds of ultrafine PVDF pristine and/or composite fibers and their roles in PM filtration.

## 2. Fabrication of Ultrafine Fibers

The targeted ultrafine fibers are not obtained from conventional melt, wet, or dry spinning processes as their end product are microfibers with dimensions of ~*O*(1)–*O*(10) micron [12,13,14,15]. “Top down” and “Bottom up” are the two most widely opted approaches for the generation of ultrafine fibers. Nanoscale drawing techniques, spinneret-based tunable engineered parameter (STEP) method, phase separation, self-assembly, template synthesis, freeze-drying synthesis, and interfacial polymerization are some of the ways of ultrafine fiber synthesis [12]. However, they lack in continuous and upscaled production of ultrafine fibers. A more developed process, called as electrospinning and relatively new nonwoven methodology, named “solution blowing” has become more popular among researchers to fabricate polymer membrane with the desired fiber size as mentioned before [13,16,17].

### 2.1. Electrospinning 

**Figure 3:**
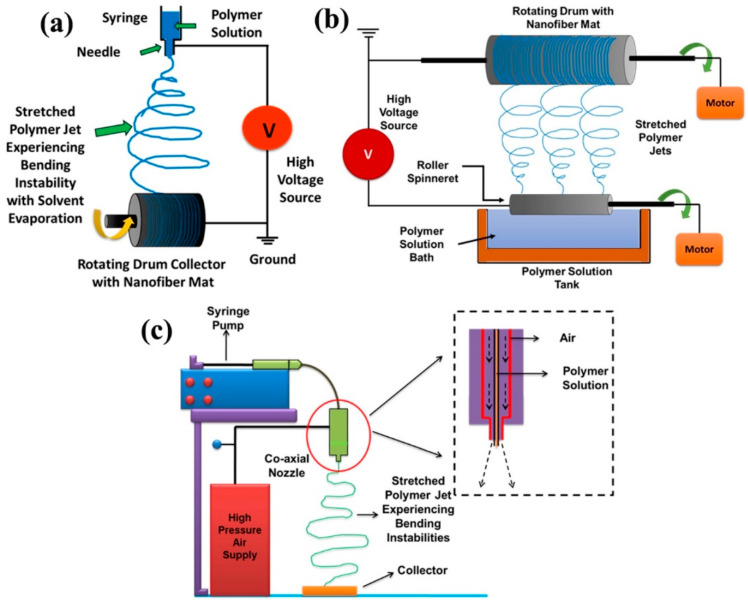
Schematic diagram of the (**a**) electrospinning process, (**b**) needleless electrospinning process using rotating electrodes, and (**c**) solution blowing process with cross-sectional view of the co-axial nozzle used in solution blowing. (**a**,**c**) are reproduced with permission from Ref. [13], copyright MDPI 2018.

Electrospinning is one of the most popular methods to create ultrafine fiber-based membrane, which has grown over the years to become a major nanotechnological manufacturing process: the well-known nanofiber membranes have seen its applications in various fields such as filtration and separation [18,19,20], tissue engineering [21], drug delivery [22], electronics [23], catalysis [24,25], thermal engineering [26], etc. The electrospinning arrangement consists of (a) a high voltage DC power supply, (b) a spinneret, and (c) a collector (as shown in Figure 3a). The spinneret is connected to the positive end of the voltage supply to be operated at 10–20 kV, while the polymer solution is pumped through the spinneret using a syringe pump with a typical flow rate of 0.05–1 mL/h. The collector works as the ground, which can be either a static plate or a rotating drum. Upon increase in the applied voltage, the ejected polymer solution drop takes the shape of a conic, and as the electrostatic field overcomes the surface tension of the solution, a thin polymer jet erupts and travels towards the collector [27,28,29]. During flight, the polymer jet narrows down and experiences several bending instabilities. At the same time, the solvent evaporates, and dry fibers get collected over the collector. Along with the abovementioned process parameters, die-to-collector distance, solution conductivity, viscosity, ambient conditions such as humidity, temperature, etc. are also critical to determine the outcome of electrospinning process [15]. Co-axial, tri-axial, needleless, bubble, and multi-jet electrospinning are some of the advanced variants of regular electrospinning process, out of which needleless electrospinning (NLES) has emerged as one of the promising techniques [30].

NLES technique is the most industrially viable of the abovementioned electrospinning variants, which overcomes the difficulties such as limited productivity, clogging of needles, multi-jet interaction in multi-needle spinning, dripping of polymeric solution, etc., encountered in conventional process [31]. It uses the technique of fabrication of nanofibers from open liquid surface, which bestows significant importance on the choices of spinneret for regulation of the final morphology of electrospun fibers [31,32]. Although the process is as old as several decades, it was only in early 2000 when it gained the popularity from work conducted by Yarin and Zussman [33] where they introduced magnetic field for generating spikes on liquid surface from which polymer jets erupted to be spun into nanofibers. Needleless spinning using rotating cylindrical spinneret has most extensively utilized NLES technique, patented under the name of “Nanospider” technology (c.f. Figure 3b [34]. However, rotating spinnerets can also be disc, ball, or spiral shaped, which greatly affect the distribution of electric field and thus the coarseness or fineness of the spun membrane [32]. Nanofibers obtained from NLES are usually of finer diameter than conventionally ES fibers, and they can be directly fabricated from polymer melt with high potential of usage in the fields of biomedical engineering [31,32].

### 2.2. Solution Blowing 

Solution blowing or solution blow spinning (SBS) technique is a rather new nonwoven process, which is alike meltblowing. The difference is that the latter deals with polymer melt and produces microfibers, whereas the former uses polymer solution and is capable of producing nanofibers [16,17]. In this process, a viscous polymeric jet gets ejected from a co-axially placed nozzle (core) at a flow rate of ~10 mL/h and compressed air (30–40 psi and ~100–200 m/s) [17] stretches the polymer jet to fabricate fibers where the jet experiences vigorous bending and flapping along with rapid solvent evaporation (c.f. Figure 3c). For solution blowing, the critical factors are the solution viscosity, the capillary forces, and the polymer relaxation time, which also serves as the basis of other extrusion processes as well. However, in this technique, the utmost importance of Deborah number (De) is undeniable as De signifies the ratio of polymer relaxation time vs. process time [35,36]. In many cases, it has been seen that researchers have mostly considered a semi-dilute to concentrated polymer solution, greater than overlap concentration (c*) for solution blowing, leading to an entangled situation and enhanced relaxation time, which also stabilizes jet to avoid capillary instabilities [36]. This yields higher De or more elastic stretching of the polymer jet leading to finer fiber fabrication [16]. 

Apart from the abovementioned manufacturing techniques, electroblowing (EB) or blowing-assisted electro-spinning is also a novel technique, which bears the fruit of solution blowing process, assisted by application of an external electric field. Apart from the stretching imparted by the strong air jet, the electrostatic pull adds additional thinning of the polymer jets to produce ultrafine monolithic fibers [37] or core–shell nanofibers [38].

## 3. Filtration of PMs Using Ultrafine Fibers

### 3.1. Measure of Performance of Air Filters

There are several factors, such as filter thickness, fiber diameter (d_f_), basis weight (grams per square meter or GSM), filter packing density (α, α = 1 − ε, where ε is the porosity), that affect the filtration efficiency of a fibrous filter. The total collection efficiency (η) of a fibrous filter is given by Equation (2) [39,40]

(2) $$\eta =1-\frac{C_{\mathrm{down}}}{C_{\mathrm{up}}},$$

where C indicates to pollutant concentration in the upstream (up) and downstream (down) across a filter membrane. Meanwhile, another factor that plays a key role in determination of filter performance is the differential pressure or pressure drop (∆p) across the filter. Ideally this should be as low as possible, to allow a continuous and seamless filtration of particles without much difficulty, say while breathing through a face mask, where the pressure drop is particularly important aspect. In a conventional melt-blown fibrous filter or spunbond-meltblown combination membrane, such as in case of N95, the microfibers aid in an increased air resistance and, consequently, result in high pressure drop. The mechanism of such is discussed later in this section. It has been observed that despite high efficiency, N95 face masks reduce ventilation, cardiopulmonary exercise capacity, and comfort and lead to dizziness, reduced oxygen supply, and itchiness to wearer [41]. Studies have shown that wearing N95 or surgical masks has resulted in decrease in VO_2max_ emission as well as reduced ventilation, due to steady decrease in breathing frequency [42]. This added breathing resistance in the upper respiratory tract may also lead to disruption in lung functionality as well as increase the risk of cardiac arrest, as the study suggests [43,44]. The high value of ∆p negatively impacts the filter’s viability and lowers the quality factor (QF), another significant performance indicator for a filter medium [45], given by Equation (3)

(3) $$\mathrm{QF}=\frac{-\ln(1-\eta )}{\Delta p}.$$

### 3.2. Mechanism of PM Filtration

**Figure 4:**
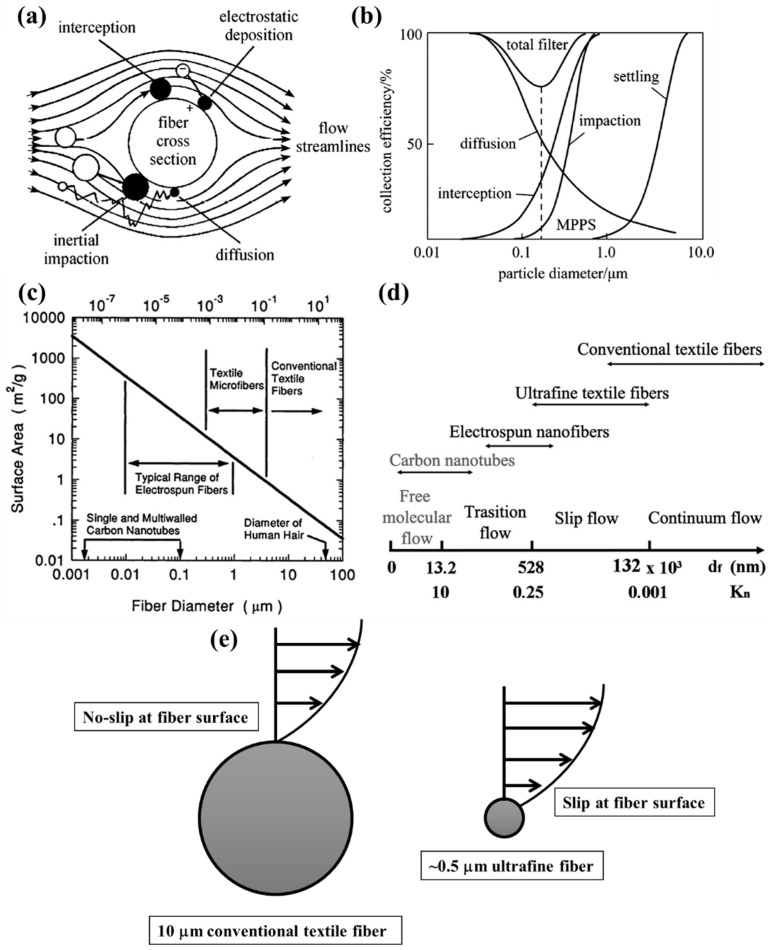
(**a**) Mechanisms of particulate matter filtration by fibers and (**b**) dependence of collection efficacy in each of the modes of capture based on particle size. Reproduced with permission from Ref. [45] Copyright Elsevier 2012. (**c**) Fiber diameter vs. surface area, (**d**) nature of flow of fluid around fiber, and (**e**) effect of reduction in fiber diameter upon slip flow. Reproduced with permission from Ref. [46]. Copyright Wiley VCH 2014.

A fibrous filter, when experiences air flow containing PMs, impedes the flow of air and the PMs get captured by the media (at surface/at depth) by several possible mechanisms. Depending on the interaction of PMs with the fibrous architecture of the filter medium, several types of filtration mechanism prevail, such as interception, impaction, Brownian diffusion, gravitational settling, and electrostatic forces of attraction play the major roles in particle capture as indicated by Figure 4a [46]. As it can be seen from Figure 4b, the increment in PM diameter dictates the capturing mechanism. At larger particle size (>1 μm), inertial impaction dominates over interception or Brownian motion, taking into consideration an assumption that the particles present in gas stream do not change its flow around the fiber, despite the fact that near fibers fluid streamlines deviate [47,48,49,50]. A particle with a diameter of ~2 μm, whose Stokes number St is > 0.5 (St = particle response time/characteristic time scale of flow), will mostly experience inertial impaction near a microfiber for a general flow velocity of ~10 cm/s. Smaller particles move along streamlines because of negligible inertia and, in such cases, when the streamline deviation is within particle radius from the fiber surface, interception becomes dominant aided by van der Waals attractive force.

As it can be seen in Figure 4b, when the size of the particle is ~0.1 µm, Brownian diffusion becomes the most feasible mechanism of entrapment. It follows the principle of concentration gradient on two sides of the filter, following Einstein’s relation as given by Equation (4) [47,48].

(4) $$D=\frac{\mathrm{KTC}}{6\pi a\eta },$$

where D stands for diffusion coefficient, which is directly dependent on the ratio (C/a), C being the Cunningham–Knudsen–Weber–Millikan drag factor, and a is the particle radius [47]. With decrement of fiber radius, deposition due to diffusion starts to predominate. However, along the discussion that with increment in particle size, inertia-based entrapment of PMs increases in fibrous filters and with reduction in size, diffusion becomes dominant, it is only intuitive that there must exist a particle size for which mechanical/pure hydrodynamical filtration faces a minimal capturing of particles, even for one of the most efficient HEPA filters. These particles are commonly called as most penetrating particles (MPPs) and can easily be identified in Figure 4b. 

The enhancement of fibrous filter’s efficiency via electrostatic force is studied for quite some time. It has been seen that for particle as small as 50 nm, surface charge plays an important role in the increment of electret filter’s efficiency, whereas about 200 nm particles get mostly caught due to polarization [48]. Penetration of particles is extremely low when filter charge is high, generating Columbic interaction between particles and filters while converting uncharged particles into dipoles and being henceforth attracted by polarization forces [49]. Columbic force operates over a distance often greater than that of van der Waals forces, which leads to capture of submicron particles of higher diameter by charged electrets. However, the particle penetration increases as soon as the quasi-permanent dipole charges start decaying. 

### 3.3. Collection Efficiency of Fibrous Filters and Emphasis of Ultrafine Fibers

The fluid flow streamlines get deviated near a cylindrical object when the streamlines are obstructed by the cylinder in windward direction. Generally, the deviation is of the order of cylinder diameter. For fibrous filters, the same phenomenon is bound to happen during air filtration when forced air is obstructed by the fibers. However, because of presence of other neighboring fibers, the flow field may get distorted significantly than a single fiber. Of course, the efficiency of single fiber is a function of the flow field. The classical fibrous filtration theory of single fiber follows Kuwabara–Happel single cell flow field consideration for array of parallel circular cylinders, which evolved from potential flow model by Albercht and Lamb’s viscous flow model [51,52]. Kuwabara hydrodynamic factor (Ku) was introduced to characterize the effect of fluid interference on the fibers, as stated in Equation (5) [51,53,54,55].

(5) $$\mathrm{Ku}=-\frac{\ln\alpha }{2}-\frac{3}{4}+\alpha -\frac{\alpha^{2}}{4},$$

where α stands for fiber packing density in filter. 

But this theory holds true for majorly conventional textile fibers, whose size range vary from 5 to 100 μm or even greater (c.f. Figure 4c). In these cases, the basic consideration of the theory is no-slip condition at fiber surfaces. Hence, continuum flow model can be a safe assumption. However, the situation calls for attention when the fiber diameter becomes smaller than the abovementioned or comparable to molecular mean free path. Nature of flow around fibers can be dictated by Knudsen number (Kn_f_) = λ/r_f_, where λ is the mean free path (65 nm at 20 °C and 1 atm pressure) and r_f_ is the fiber radius. The flow of gas around a single fiber in a filter can be differentiated into different flow regimes based on the value of Knudsen number as illustrated in Figure 4d [56]. When fiber radius attains a value lower than 1 μm, Kn_f_ becomes significant enough to consider slip flow at the fiber surface. With Kn_f_ values of 0.001–0.25, the slip flow regime of gas around the filter becomes predominant as evident from Figure 4d. The slip of molecules on the fiber surface results directly in lowering of drag force on the fibers, in comparison to the non-slip flow. This indicates lowering of pressure drop, (∆p) which is commonly observed in case of nanofibrous filters [56,57]. 

The usage of ultrafine fibers in filter surface indicates a decrease in pore sizes, which can be directly related to the effect of slip flow on the fiber surface by a relation, as stated in Equation (6) [53].

(6) Ck=[(2k1dfμ)/(Reln(k2)db)],

where C_k_ represents the drag coefficient_,_ constants k_1_ and k_2_ represent shape factor and arrangement of fibers, respectively, d_f_ is the diameter of fibers, Re=ρvdf/μ is Reynolds number, d_b_ is the distance between the neighboring fibers, and μ is the viscosity coefficient of air. The inhomogeneity in fiber alignment in ultrafine fibrous filters lead to a correctional term, namely, equilibrium factor (τ) determined as τ = d_f_/d^2^, where d is assumed to be the equivalent diameter of pores. This equilibrium factor helps to identify the relation between porous structures and reduced drag force. For ultrafine fibers, the optimum pore size is important for reduction in air flow resistance and an increase in slip effect [57]. Considering Kuwabara’s flow field, the effect of gas slip upon the pressure drop of fibrous filter can be estimated by Equation (7) [39].

(7) $$\frac{\Delta p}{L}=\frac{U\mu \alpha L\left(1+1.996Kn_{f}\right)}{d_{f}{}^{2}\left(Ku-\alpha +0.5\left(1+\alpha^{2}\right)\right)}.$$

Alternatively, a more generalized relation was found by Davies for calculating pressure drop given by Equation (8) [47].

(8) $$\frac{\Delta p}{L}=\frac{64\mu U\alpha^{3/2}}{d_{f}{}^{2}\left(1+56\alpha^{3}\right)}.$$

In both the equations, U represents face velocity and L signifies the membrane thickness. Lowering of pressure drop leads to an increase in QF of the filter, as evident from Equation (3), which is an indication of its efficient performance of filtration of submicron particles. De facto Davies model directly predicts the inverse relation between pressure drop and fiber diameter, when other factors are constant, especially $\Delta p\propto 1/{d_{f}}^{2}$. This automatically puts the ultrafine fibrous filters in advantageous position compared to their meltblown/spunbond counterparts, especially when the pore size can be tailored as mentioned by Ref. [47].

The collection efficiency of ultrafine fibrous filters is mainly dependent upon the diffusion of the particles, especially when they are <0.1 μm in size, deviating from streamline or by the interception of the comparatively bigger particles (>0.5–1 μm) at the fiber interface, already discussed above. The diffusion-based separation is chiefly determined by a characteristic parameter called Peclet number (Pe) $\mathrm{Pe}=Ud_{f}/D$. It clearly hints at the fact that a lower Peclet number, indicative of stronger diffusive nature, can also be obtained by decrement in fiber diameter. The Reynolds number near ultrafine fiber becomes ~*O* (0.01–0.1), where creeping flow is expected, allowing stronger diffusion, less perturbed by convection. The single-fiber filtration efficiency (SFFE) based on diffusion can be approached using towards boundary layer assumptions. Langmuir proposed to calculate filter efficiency for diffusion ($\eta_{d}$) by considering effective thickness of fluid layer, where the penetration time for particles to fiber surface is similar to the diffusion time of particles through the filter [58]. Later, it was proposed that along with $t_{d}\sim \pi d_{f}/D$ (where t_d_ = time for diffusion), a dimensionless parameter (Ω) for drag must be used for prediction of diffusion efficiency, which is a function of pressure drop across a filter of certain thickness [59]. Furthermore, the effect of neighboring fibers as well as slip flow regime was taken into consideration. Liu and Rubow et al. (1990) [60] calculated $\eta_{d}$ for single fibers using Equation (9)

(9) $$\eta_{d,\mathrm{Liu}}=1.6{\left(\left(1-\alpha \right)/\mathrm{Ku}\right)}^{1/3}Pe^{-2/3}C_{d},$$

where C_d_ is a slip correction factor given by Equation (10)

(10) $$C_{d}=1+0.338K_{n}{\left[\left(1-\alpha \right)\mathrm{Pe}/\mathrm{Ku}\right]}^{1/3}.$$

However, Equation (9) was later modified by Payet as it was found that for lower Pe, the expression may predict >100% efficiency. Therefore, $\eta_{d}$ is given by [52]

(11) $$\eta_{d}=1.6{\left(\left(1-\alpha \right)/\mathrm{Ku}\right)}^{1/3}Pe^{-2/3}\left(\frac{1+0.388Kn_{f}{\left(\frac{(1-\alpha )\mathrm{Pe}}{\mathrm{Ku}}\right)}^{1/3}}{1+\eta_{d}{}_{,\mathrm{Liu}}}\right).$$

Similar to diffusion, the efficiency of single fibers for separation via interception ($\eta_{\mathrm{IR}}$) was calculated with several approaches. Interception coefficient (R) ($R=d_{p}/d_{f}$, where d_p_ is particle diameter) was considered as the basis of efficiency measurement where flow rate coincides with fiber volume fraction [46]. There was hardly any parity found between theoretical SFFE for interception and the experimental results, until effect of neighboring fibers, packing density, limiting streamlines, and slip flow were taken into consideration [52]. Lee and Liu et al. (1982) [61] proposed interception efficiency ($\eta_{\mathrm{IR}}$) given by Equation (12)

(12) $$\eta_{\mathrm{IR}}=\left(\left(1-\alpha \right)/K_{\mathrm{Ku}}\right)\left(R^{2}/\left(1+R\right)\right).$$

Liu and Rubow et al. (1990) [60] modified Equation (12) considering slip flow regime and introduced a correction factor, C_r_, as given in Equation (13).

(13) $$\eta_{\mathrm{IR},\mathrm{Liu}}=0.6C_{r}\left[\left(\left(1-\alpha \right)/K_{\mathrm{Ku}}\right)\;\left(R^{2}/\left(1+R\right)\right)\right],$$

where C_r_ is

(14) $$C_{r}=1+\left(1.996K_{n}/R\right).$$

Overall single fiber efficiency E can be expressed through Equation (15) [39]

(15) $$E=1-(1-\eta_{\mathrm{IR}})(1-\eta_{d}).$$

Though, inertial impaction is not a dominant phenomenon for capture of particulates by ultrafine fibers, it has been found that SFFE via inertial impaction is directly impacted by drag force, which is negligible in the transition and slip-flow region. Generally, St number is used for defining the particle movement, which is given by the ratio of particle relaxation time vs. hydrodynamic time [62,63].

Hence, in case of ultrafine fibers and for particles with size of ≤1 μm, total filter efficiency (η) for a fibrous filter is generally calculated using Equation (16) [39].

(16) $$\eta =1-\exp\left[-\frac{4\alpha \mathrm{EL}}{\pi d_{f}\left(1-\alpha \right)}\right].$$

In comparison to a spunbond or meltblown fibrous filter, a filter made of ultrafine fibers will have much higher number of fibers at a particular basis weight (GSM), providing higher opportunities for particle to dock. Thus, the filter efficiency (η) will show a steady increase with decrease in fiber diameter, mostly contributed by interception and diffusion of the particles in slip-flow regime. Thus, ultrafine fibers can advantageously be used for synthesis of ultra-low GSM filters, without negatively impacting the overall filter performance. 

### 3.4. Some Examples of Ultrafine Fiber-Based Filters in Air Filtration

**Figure 5:**
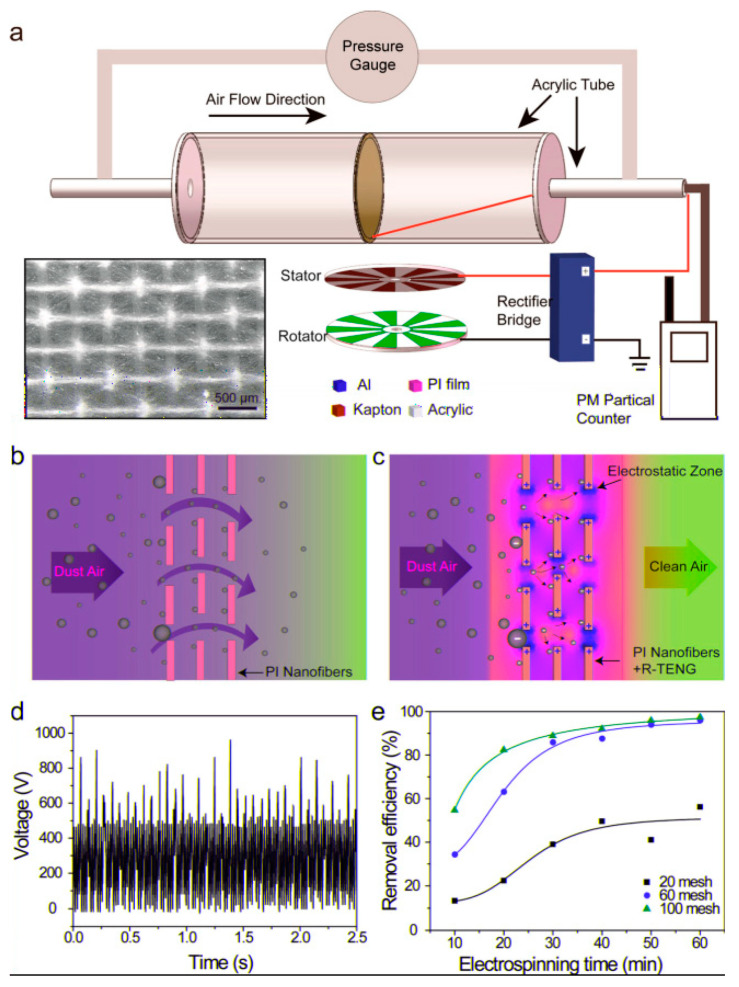
(**a**) PM removal efficiency measurement device illustration, schematics of the filtration mechanisms of the filter (**b**) without TRNG and (**c**) with TRNG; (**d**) rectified voltage of the TRNG; and (**e**) comparison of the PFE (%) of air filters with different meshes and electrospinning time. Reproduced with permission from Ref. [73]. Copyright ACS publications 2017.

The ultrafine fibers chiefly enhance the collection of PMs at surface scale in comparison to the depth filtration of conventional fibrous filters. Electrospun polyimide (PI) nanofibers with diameter of ~300 nm showed excellent PM_2.5_ retention capacity (>99%) [64]. The polyimide nanofibers demonstrated high thermal stability between 25 and 370 °C and unperturbed PM removal capacity within this range. Polyimide nanofiber membrane with an average fiber diameter of 380 nm and a pore size of 5.3 µm was manufactured by multi-jet solution blow-spun method using polyamic acid (PAA) [65]. Similar to previous work, these membranes exhibited excellent thermal stability at temperatures up to 420 °C and high filtration efficiency for PM_2.5_, as good as 99.73% at aerial density of 6.61 g/m^2^ with a pressure drop of 126 Pa, when tested at room temperature and at flow rate of 32 L/m. The nanoparticles of various size range (20–600 nm) were used for these measurements. In a separate work, “bead on string”-based PAN nanofibrous filter membrane was developed via electrospinning using a 5 wt% solution with an average fiber diameter of 70 nm and an average bead size of 249 nm [66]. Such structure was obtained using an optimized polymer concentration and relative humidity condition. Such ultrafine nanofibers were extremely useful in filtering NaCl/paraffin aerosols (solid/oil) of mass mean diameter 300 nm up to 96% at a meagre pressure drop of 14.6 Pa. The mechanism of particle capturing was predominantly Brownian diffusion, which was weakened as the velocity of the gas flow was increased, yielding lower efficiency [66]. Glass particle doped (up to 5%) solution-blown PA-6 nanofibers of an average fiber diameter of 150–200 nm exhibited an increase in air permeability by 200% when compared with untreated PA6 nanofibers. The untreated and treated PA-6 nanofibers both demonstrated PFE of ~>99% for PM_2.5_, however, the latter reduced the pressure drop almost to 50% than the former [67]. Very recently, a unique semi-interpenetrating nanofibrous aerogel (IPNFA) structure has been investigated for capturing variety of polydisperse fine particles (PFP) through its sponge-like open-pored hierarchical structure, mimicking loofah [68]. An IPNFA polyamide-imide (PAI) aerogel structure was developed through freeze casting of electrospun PAI fibers, followed by cross-linking through incorporation of bismaleimide (BMI) flexible monomers. This structure filtered PFPs through cascade filtration, using gradient of the particulates as the principle. The topmost layer captured PM_3_, the middle layer filtered PM_1.0_ and PM_0.5_, whereas the bottommost layer selectively captured PM_0.5_ and PM_0.3_, with a filtration efficiency of about 99.98% for each of the PFPS, exceeding the requirement of 99.97% for HEPA filter. A sustainable alternative to synthetic nanofibrous filters was proposed through fabrication of cellulosic nanofibers (CNF, *not to be confused with carbon nanofibers*), by nanofibrillation of pulps from various tree species. A CNF/PES composite electrospun nanoweb with nanofibers ranging from 67 to 89 nm, supported by a PET meltblown substrate, has been found to show significantly high QF of about 0.083 Pa^−1^ compared to the PES nanoweb alone with a pressure drop of 26 Pa for 0.4 μm particle at a face velocity of 5.3 m/s [69]. The enhanced PFE was due to the presence of polar hydroxyl groups at the surface increasing its van der Waals forces of interaction with the PMs. At the same time, the reduced pressure drop was seen owing to the non-zero velocity at fiber surface or no-slip condition, which is prevalent at these fiber sizes. Ultrafine electrospun PVA nanofibers doped with cellulosic nanocrystals (CNC) also exhibited a stable filtration efficiency of 99.97% for PM_2.5_, even after 5 filtering cycles with a negligible rise in pressure drop from 60 to 77 Pa [70]. Electrospun nanofibers, such as PAN and PES, when functionalized with additional fillers, such as grapheme oxide (GO) and metal organic frameworks such as UiO-66-NH_2_(Zr), led to an increased adsorption efficiency for PM_2.5_, carbon dioxide, and other VOCs, due to increased interaction between particulates and functional groups of the additives [71,72]. Ultrafine electrospun nanofibers embedded with silver (Ag) nanoparticles are also known to exhibit additional antimicrobial efficiency, alongside increased PFE [73]. Triboelectric nanogenerators (TRNG)-enhanced PI-based nanofibrous filter demonstrated PFE of about 90% at 33.6 nm particle size. Along with mechanical modes such as inertial impaction and interception, the electrostatic filtration boosts the overall PFE for particles with size of <100 nm (as observed from Figure 5c). The TRNG principle used by this system also mitigated the risk of ozone generation [73]. Biodegradable filters made of silk protein, called as silk nanofibrous air filters (SNAF), were also developed to separate PM_2.5_ with a PFE of 97% and a QF of 0.3 at particle concentration of 444 μg/cm^3^ [74]. Similar efficiencies were observed in silver (Ag)-doped keratin/nylon-6 nanofibrous composite membranes, where the average fiber sizes varied between 155 and 293 nm for various combination of silver doping and keratin concentration [75]. This combination demonstrated significant antimicrobial efficiency against bacterial species such as*S. aureus* and *E. coli*. Besides, other biopolymers such as zein polypeptide [76] or deacetylated chitosan [77] have exhibited high particulate removal efficiency even for PM_0.3_, when converted into nanofibers. Their efficient performance as air filters were due to the active sites present on suitably grafted fiber surfaces, which can neutralize harmful toxins by reacting with them [78]. Hierarchical dual structure of 2D nano-net combined with a 3D fibrous scaffold, manufactured from electrospun PAN/tetra-n-butyl ammonium chloride (TBAC) demonstrated excellent PM_0.3_ removal efficiency (>99.99%) and had a QF of 0.1 PA^−1^. The ion–dipole interaction between the PAN and TBAC led to charging of the filter surface, with tunable pore size (<300 nm), high porosity (93.9%), and low packing density with long-term filtration promise for PM_2.5_ [79].

## 4. Ultrafine PVDF Nanofibers for PM Removal 

PVDF is a non-reactive thermoplastic fluoropolymer, which is produced by the polymerization of vinylidene difluoride unit. PVDF and its copolymers, mostly trifluoroethylene (PVDF-TrFe) derivatives were found to be simultaneously piezoelectric, ferroelectric and pyroelectric, prior to the similar traces found in any other polymers such as PLA, polyurethane (PU), cellulosic, polyamides, and others. Since then, PVDF has gained enormous importance as an alternative material to synthesize energy storage devices replacing batteries, tactile sensors, and, recently, electret air filters.

### 4.1. Structure and Properties of PVDF

**Figure 6:**
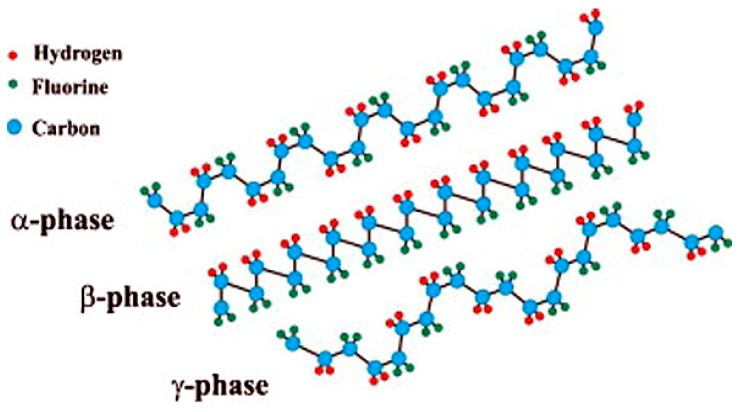
Basic polymorphic structures of PVDF polymer. Reproduced with permission from Ref. [81]. Copyright Elsevier 2014.

Polyacetylene and PVDF are among the initial identified specimens of conjugated organic polymers with high magnitude of nonlinearity and architectural flexibility [80]. Polymers initiate conductance, when the net dipole moment of the polymeric system along the chain axis attains a non-zero value at ground condition, under the effect of crystallographic phase transformations and external electric field. PVDF possesses a backbone of [-(CF_2_-CH_2_)_n_-], where the C-F and C-H bonds induce a dipole moment of about 5–8 × 10^−30^ C-m due to strong electronegativity of fluorine atom compared to carbon or hydrogen atom [81]. PVDF is semi-crystalline in nature with a crystallinity percentage of about 50–70%, where the lamellar crystals grow out of spherulitic structures, leading to a sandwiched structure of crystalline and amorphous regions [69]. PVDF exists in five interconvertible polymorphic structure in the form of α- and δ-(${\mathrm{TGTG}}^{\prime }$), β-(TTT), γ, and ε-($T_{3}GT_{3}G^{\prime }$) (T for trans and G for gauche) when subjected to various mechanical, thermal, or electrical processing. Out of these, α-, β-, and γ- are the most researched ones and their structures are shown in Figure 6 [81]. Out of all these phases, TTT phase possesses the highest dipolar moment per unit cell and hence is one of the most go-to material for sensors, actuators, separators, batteries, filters, even in biomedical field as well [82,83]. The conversion of α- to β-form is generally caused by mechanical stretching from melt under high pressure or electric field or quenching, etc. [84]. The overall trans confirmation of the molecular chains in the β-phase combined with specific polarization in the crystallites result in the highest piezoelectric effect, with additional ferroelectricity and pyroelectricity [80,85].

The β-phase of PVDF shows stable remnant polarization due to stability of nuclei consisting of co-operative dipole–dipole interaction as well as reduction in internal fields by small, non-reversed domains at the boundaries of crystallites, minimizing energy [85]. Thus, PVDF and, especially, β-PVDF might hold an advantage over other polymers used in manufacturing fibrous filters due to its additional contribution as an excellent electret.

### 4.2. Fabrication of Ultrafine PVDF Fibers

Electrospinning or solution blowing result into varying diameters of PVDF fibers, *O* (10 nm to 1 μm). However, for sake of brevity and for this review, we will keep the discussion limited to the previously mentioned ultrafine range only. The polymorphic transformation of α phase to β phase in the crystallite structure of PVDF happens during ultrafine fiber synthesis due to the uniaxial stretching imposed during electrospinning and solution blowing. 

#### 4.2.1. Synthesis of Ultrafine PVDF Fibers by Electrospinning

**Figure 7:**
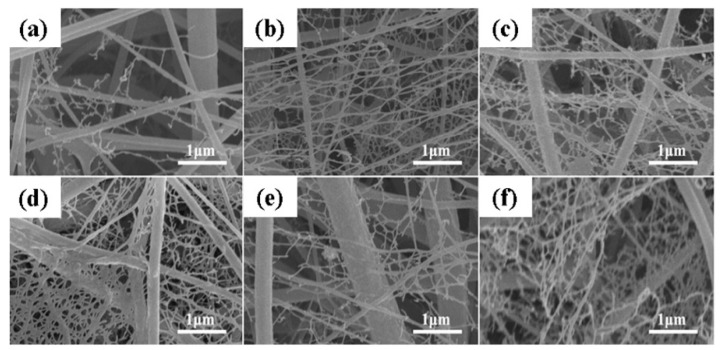
FE-SEM images of nanofibers obtained from PVDF-TBAC (0.1 mole/L) membranes formed with different process parameters: (**a**) 20 kV, 15 cm, 1 m/h, (**b**) 25 kV, 15 cm, 1 mL/h, (**c**) 30 kV, 15 cm, 1 mL/h, (**d**) 25 kV, 10 cm, 1 mL/h, (**e**) 25 kV, 20 cm, 1 mL/h, and (**f**) 25 kV, 15 cm, 2 mL/h. Reproduced with permission from Ref. [89]. Copyright Elsevier 2016.

PVDF and its derivatives are electrospun into different kinds of ultrafine fibrous structures, finding diverse applications, because of their piezoelectricity properties. N,N-dimethylacetamide (DMAC) and acetone in 1:1 mixture was used as solvents to fabricate PVDF nanofiber-based scaffold with average fiber diameter of 352.9 nm [86]. The process parameter included solution concentration, applied electric field, and collector rotation speed. The effect of three solvents N,N-dimethylformamide (DMF), N-methylpyrrolidone (NMP), and dimethyl sulfoxide (DMSO), along with acetone in 60/40 ratio (acetone, 40 percent), was observed for beadless ultrafine PVDF nanofibers fabrication, at a polymer concentration of 12 wt% [87]. The least nanofiber size (415 nm) (with ~90% β-phase fraction) was obtained for DMF/acetone mixture, with various other processing parameter. PVDF-ZnO nanofiber of dimension ~400 nm, was fabricated via electrospinning using DMF as a solvent for both piezoelectric and photoconduction applications [88]. In a separate work, PVDF tree-like nanofiber web (PVDF-TLNW) was fabricated using a one-step electrospinning process where the fiber size varied from 5 to 500 nm in diameter [89]. The main solvent was DMF, and tetrabutylammonium chloride (TBAC) was used as an additive. The nanofibers mimicked natural tree-like structure with over 50% of branch-like fibers (see Figure 7). Experimental parameters of electrospinning have an enormous effect on the morphology of the PVDF nanofibers [90]. An increase in ratio of DMF:acetone, i.e., with increment in DMF concentration in the solvent system for PVDF, has been found to generate beaded nanofibers, due to the lowering of solvent vapor pressure leading to incomplete solvent vaporization and limited stretching of polymeric chains. Study of additional effects of relative humidity (RH) during the electrospinning of PVDF system, at constant DMF/acetone ratio, revealed that with an increase in RH from 0% to 50%, the ultrafine fiber size increased from 130 to 240 nm [90]. Similarly, the effects of the process parameters of electrospinning upon the content of β-phase in PVDF nanofibers have also been experimentally concluded [91,92], which are calculated as per Equation (17)

(17) $$F\left(\beta \right)=\left(\frac{X_{\beta }}{\left(X_{\alpha }+X_{\beta }\right)}\right),$$

where X_α_ and X_β_ represent crystallinity percentages in α- and β-phases, respectively. Uniaxial stretching of polymeric jet (such as in electrospinning) results in an oriented dipole system in PVDF polymer chain, thus effecting conversion of α- to β-phase. This conversion has been found to be directly proportional to high voltage supply, low feed rate, and fine gauge of syringe needle. Free-standing PVDF nanomembrane using circular electrodes was attempted as a novel method to develop multifunctional filters for dust filtration coupled with humidity blocking [93]. In case of the circular electrodes, nanofibers were collected on circle electrode collector, which was transferred to substrates using Si wafers, glass, polystyrene, etc.

#### 4.2.2. Synthesis of Ultrafine PVDF Fibers by Solution Blowing

As mentioned above, solution blowing has become relevant in nanofiber manufacturing methodologies in last decade from its inception in 2009 by Medeiros et al. (2009) [94]. Solution blowing was used for generation of UV-crosslinked PVDF nanofiber membrane containing ultrafine fibers in the range of 40–140 nm, with an average of 100.28 nm using DMF as a solvent. Briefly, 2,4,6-trimethyl benzoyldiphenylphosphine oxide (TPO) was used as the photo initiator for UV-irradiated in-situ cross-linking of PVDF nanofibers with trimethylolpropane tri-acrylate (TMPTA) as a cross-linker [95]. Addition of TPO decreased viscosity of the solution-blown polymeric jet, which resulted in a decrease in the average fiber diameter from 121.48 to 87.42 nm, with an increase in TPO concentration (w/w) from 1% to 7%, with a simultaneous decrease in porosity from 72% to 64%. Solution blow spinning (SBS), also known as solution blowing, was conducted using a commercial airbrush to generate PVDF nanofibers [96]. Concentration of 20 wt% in DMF as a solvent, 5 bar of gas pressure, and 20 cm of tip to collector distance (TCD) were the optimized conditions to prepare ultrafine nanofibers with a diameter of ~138 nm. High flowrate at the tip of airbrush is one of the primary factors that impeded formation of a proper viscoelastic jet and subsequently formation of nanofibers. Solution-blown PVDF nanofibers were sandwiched between PVA-PEDOT/PSS layer and an aluminum foil to generate a flexible piezoelectric nanogenerator [97]. The PVDF nanofibrous membrane served as the basic component of generator, with an average thickness of 50 µm and an average fiber diameter of 400 nm. The high piezoelectric performance of the same is attributed to the significant β-phase crystallization and conversion of residual α- into β-phase during stretching of the jet, where high degree of stretch results in rearrangement of the crystallites into stable β-phase at working temperatures below 80 °C, ascertained by FTIR and Raman spectral studies [97,98]. Effect of binary solvent of DMF and acetone in solution blowing of PVDF nanofibers was also investigated. The increasing proportion of acetone decreased the viscosity of the jet, which led to syringe blockage. The optimum concentration of DMF/acetone was found to be 8/2 where beadles nanofibers of 394 nm was obtained with β-phase fraction of 85% [98]. LiCl-ZNO-PVDF humidity sensors are also being manufactured using solution blowing technique for PVDF in DMF solution, using LiCl and ZNO as dopants, obtaining nanofibers in the range of 400–500 nm. Electrospinning became an inappropriate option in this case, owing to the presence of conductive LiCl molecules, which disabled jet stretching due to charge repulsion [99].

### 4.3. Ultrafine PVDF Fibers for Filtering Air-Borne PMs

Conductive polymers such as PVDF, polyurethane, polyvinylpyrrolidone (PVP), etc. have recently been explored in the fields of electret air filters and are found to be promising, owing to high charge stability, low conductivity, effective electrostatic interaction between surface–volume charges and PMs. To efficiently capture PM_2.5_, PVDF nanofibers, with diameter in the range of 200–400 nm, were electrospun and collected as a single layer upon conventional melt-blown fibrous meshes of polyester and nylon with mesh number ranging from 60 to 120 [99]. The PVDF dissolved in DMF was electrospun through multi-needle setup containing silver nitrate as a dopant, which an imparted antimicrobial efficiency of about 99.99% to the nanofiber-coated air conditioner filter. Such layered membrane exhibited the highest filtration efficiency of 95.5% at PM_2.5_ when produced at a rate of 8 mm/s and an efficiency of 98.23% when produced at a rate of 4 mm/s with polyester and nylon substrates of mesh number 80. Li et al. (2016) [89] showed that the TLNW membrane with variation in diameter of main and branch-like fibers allowed efficient capturing of 0.26 μm at 32 L/m air flow rate with 99.999% efficiency at meagre 124 Pa pressure drop. The branch finer fibers participated in blocking the particles as second “door keepers” apart from the main fibers due to increased surface area leading to enhanced interception of particles. The additional effect of van der Waals interaction between ultrafine (<100 nm) fibers and nanoparticles are also noteworthy in these cases, as was also pointed out earlier by Sinha-Ray et al. (2015) [16]. A similar concept was also attempted by another group of researchers [100] where one-step fabrication of PVDF branched ultrafine nanofibers with a diameter of 50 nm gave rise to PFE of 99.999% at PM_0.26_ with minimal pressure drop of 126.17 Pa. Solution-blown PVDF nanofiber membrane sandwiched between two PP layer was introduced in a separate work [101] along with other polymers such as PAN and cellulose acetate as an alternate to 3-ply surgical mask which generally fairs poorly in separation of PM_2.5_ (≤2.5 μm). Although PVDF nanofiber membrane, consisting of an average fiber diameter of 660 nm, performed poorer than PAN membrane, mostly due to large pore size and bigger nanofiber, the work showed that such an alternative to surgical masks can be a viable option. The solution-blown UV-crosslinked PVDF nanofiber membrane prepared by Liu et al. (2020) [95] was able to capture 100% of the particles with size of >230 nm when such membranes were exposed to various particles with a diameter of 200–500 nm flowing at a speed of 0.053 m/s. Such membrane efficiently utilized the ultrafine diameter of <100 nm nanofibers to synergistic effect of enhanced nanofiber–nanoparticle interaction, along with “slip at fiber surface” for reduced pressure drop. 

It has already been proven by now that using electrospinning or solution blowing, one can manufacture ultrafine PVDF nanofibers, which have immense potential in PM trapping from air [100,101,102]. Albeit the charge retention capability of PVDF nanofibers makes such membrane more lucrative to use as an electret air filter rather than a pristine nanofiber for pure mechanical separation. 

#### 4.3.1. Mono-Layer Ultrafine Electret PVDF Nanofiber Filter 

**Figure 8:**
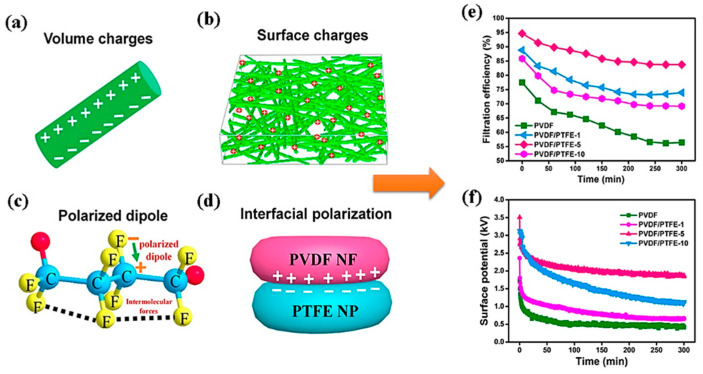
(**a**) Volumetric charges of nanofiber, (**b**) surface charges of membrane, (**c**) polarized dipole of PVDF fiber, and (**d**) interfacial polarization of PVDF NF-PTFE nanoparticle composite. The PFE and surface potential variation with time is shown in (**e**,**f**). Reproduced with permission from Ref. [106]. Copyright ACS publications 2016.

**Figure 9:**
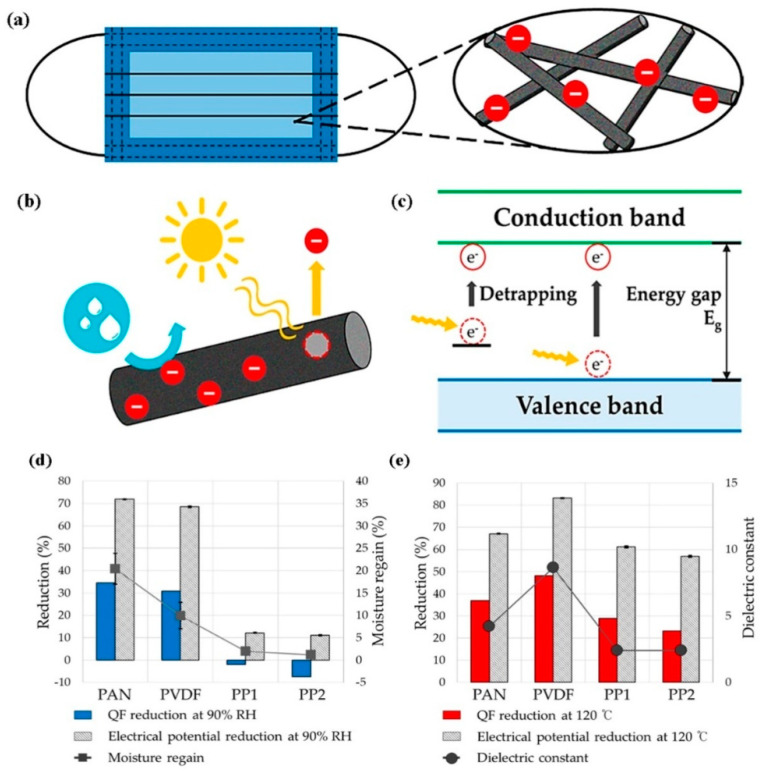
(**a**) Electret fibers applied to a filtering respirator, (**b**,**c**) electret mechanism of the filter, (**d**) quality factor reduction (%) and moisture regain (%) for different kinds of fibers at 90% RH, and (**e**) QF reduction (%) and moisture regain (%) for different kinds of fibers at 120 °C. Reproduced with permission from Ref. [102]. Copyright MDPI 2020.

An electret filter is the most effective technological intervention in mitigating the competitive pattern of efficiency increment with pressure drop rise for commercial air filters such as HEPA. Significant increase in efficiency can be observed in an electret filter at minimal increment in air flow resistance, because of additional electrostatic interaction coupled with mechanical modes of separation or PMs from air, which also aid in preparation of low GSM filters for practical purposes. Electret fibrous filters are manufactured by various techniques, such as corona discharging, triboelectrification, induction charging, and liquid contact charging [11], whereas electrospun PVDF, PP, and PU nanofibers already exhibit electret property because of sufficient surface charge traps or already polarized structure such as β-phase in PVDF [102]. The lower the diameter of nanofibers, the higher is the contribution of electrostatic mode of filtration, especially for particulates of size less than 0.1 μm. The electrostatic charges can stay on ultrafine fibers for very long times due to more stable space charges in the nanofiber mat with an increment in surface area, and in such cases, the single-fiber efficiency is higher in nanofibers compared to microfibers [103]. Electro-assisted solution blowing (EBS) was also employed to generate PVDF ultrafine nanofibers to manufacture electret filter to separate aerosol particulates with an average diameter of 0.3 µm [104]. When the electrostatic voltage was increased from 10 to 30 KV, the average fiber diameter (AFD) could be reduced by ~100 and 300 nm for 12 and 16 wt% polymer concentrations, respectively. There was also a significant rise in filtration efficiency from 70% to about 85%, with a very low rise in pressure drop of about 4 Pa. in charged filters. Upon application of isopropyl alcohol (IPA) solution on the fibrous mats, to discharge them, the filtration efficiency was lowered significantly, indicating the major contribution of electro-blown β-form of PVDF as an electret, alongside mechanical modes of capture. In a separate work conducted by Lolla et al. (2016) [105], ultrafine electrospun PVDF nanofibers with an average diameter of 190 nm from DMF/acetone was produced from 10 wt% polymer solution using trifluoracetic acid (TFA) as an additive to it. The addition enhanced bead-free fiber formation. The polarization of the fibers was done at an electric field of 2.6 kV/cm. The PFE measurement at continuous testing of 330 days revealed that the ultrafine charged membranes retained their efficiency at a much higher value than micro-fiber-based membranes of PVDF, both charged and uncharged. Wang et al. (2016) [106] (c.f. Figure 8c,d) used electrospun PVDF nanofibers with poly-tetrafluoroethylene (PTFE) nanoparticles embedded in them, where the latter participates as charge enhancer. During electrospinning, this multi-component system gets polarized due to the supply of high voltage and creates volume and surface charge (c.f. Figure 8a,b), which is also enhanced due to strong electronegativity of fluorine atoms and the interface between PVDF/PTFE interface. In addition, with increased use of PTFE nanoparticle, the PVDF nanofiber diameter reduced from 622 to 380 nm, with possible bead formation. These total factors led to enhanced PFE of 94% for optimized PTFE concentration at 0.05 wt% with pressure drop of about 18 Pa due to synergistic effect of mechanical separation and optimum surface charge potential. The lowering of PFE was concomitant with reduction in surface potential (see Figure 8e,f). The homo-electrets made from low conducting or nonpolar polymers hold stable electrical charges, but their limitation comes from the charge decay due to heat, humidity, and solvent-mediated deterioration [107]. Alternatively, the electret charge decay may happen due to emancipation of fiber-trapped charge owing to molecular agitation with scavenging effect of solvent molecules [108]. Along with that, the environmental factors such as thermal ageing, RH are also considered (see Figure 9a–c). A detailed study has been conducted to speculate the effects upon the electret performance of electrospun PVDF, PAN, and corona-charged melt-blown PPE fibrous webs for filtration of 75 nm NaCl particles [109]. It was found that under accelerated conditions of ageing at a temperature of 120 °C and a relative humidity of 90% for 48 h, the charge decay was in the order of PVDF > PAN > PPE. This was attributed to the highest dielectric constant (ε_r_) of PVDF (8.4–8.9) in comparison to 4.2 of PAN and 2.2–2.6 of PPE, which led to easy loss of charge through the small band gap, at extremely low activation energy. The filtration efficiency and quality factor of the electret media followed suit [110].

A 2D self-polarized nanonet structure obtained using electrospinning of PVDF, with fiber size of about 21 nm and surface potential of about 6.8 kV and having 86% of β-phase was employed for PM_0.3_ filtration [110]. The high porosity and mean pore size of 0.3 µm, about one-tenth of that of a fibrous web, along with heightened surface potential led high PFE (99.998%) at low pressure drop. Both sieving and electrostatic attraction played their part in nanoparticle entrapment. 

#### 4.3.2. Multi-Layer Ultrafine Electret PVDF Nanofiber Filter

**Figure 10:**
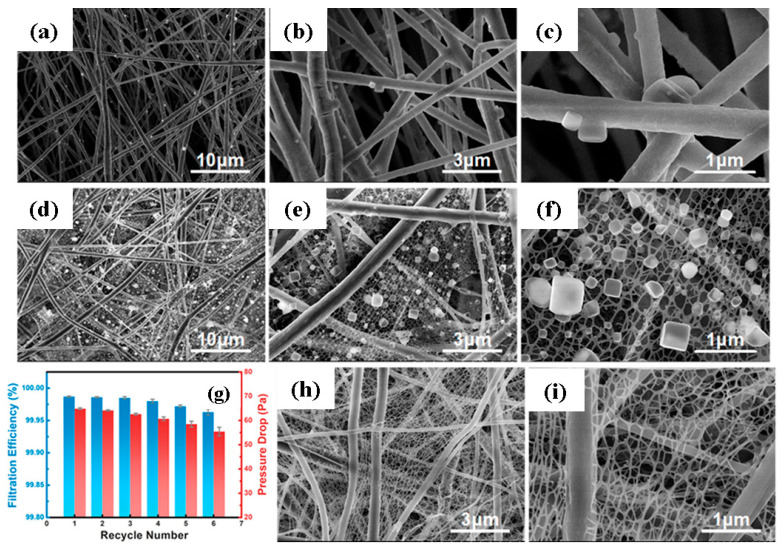
(**a**–**c**) SEM images of PVDF nanofibers, (**d**–**f**) SEM images of PVDF nanofibers/nanonets, (**g**) filtration efficiency (%) of the PVDF nanofibers/nanonets with recycle number, and (**h**,**i**) FE-SEM images of nanofiber/nanonet filters after the sixth cleaning cycles. Reproduced with permission from Ref. [114]. Copyright ACS Publications 2018.

Filtration efficiency of PVDF monolayered [111] and multilayered membrane [112] has been tried to be optimized. Electrostatically charged PVDF nanomembranes develop a dendritic structure upon collection of aerosols through electrostatic forces, leading to formation of an aerosol cake, ensuring further filtration almost with 100% efficiency, however, this results in an increase in pressure drop [57]. This further results in “skin effect”, which causes collection of more particles on face side of the filter than on the back side. Eventually, this results in shielding of electret fibers by particles, where the rise in pressure drop is more compared to PFE, and consequently, the mechanical separation of PMs controls the filtration behavior. The work conducted by Sun et al. (2020) [112] showed that multi-layered electrospun PVDF charged membranes of 3 GSM basis weight (4 layers) always performed better than uncharged one with nearly 100% PFE. The interesting finding was that the multi-layered charged membranes captured 80–250 nm particles mostly at the filter and less by the developed cake at reduced pressure drop, whereas the single layer showed exact opposite behavior. Multi-layered PVDF nanofilter decreases skin effect by allowing uniform aerosol loading on both sides of the filter, owing to dielectrophoresis, thus lowering the rate of dendrite formation. The same group of researchers used electrospun PVDF multi-layer membranes with an average fiber diameter of 525 nm for separation of non-spherical particles [113]. The authors investigated the charged PVDF filters in filtering particles with size of 20–300 nm, with special emphasis on irregularly shape particles, especially COVID-19 virus size-scale, keeping a target of PFE of ~90% at not more than 30 Pa pressure drop. It was found that with an increase in layering for both charged and uncharged filters, the filtration efficiency sharply increased, and the pressure drop decreased. The most penetrable particle size (MPPS) decreased to 75 nm for charged PVDF fibers from 150 nm for uncharged fibers. The efficacy of mechanical separation with interception and Brownian diffusion was prevalent for both the cases, where the former could enjoy net 50–64% of entrapment contribution arising due to dielectrophoretic phenomenon, which was boosted by increasing the number of layers [114]. A 2D, layer-by-layer nanofiber/nanonet structure of electrospun PVDF membrane was effectively utilized for efficient removal of PM_0.26_ at an PFE of 99.985% and pressure drop of <70 Pa for an air flow rate of 32 L/m [114]. The nanonet fabrication was promoted via anionic surfactant sodium dodecyl benzenesulfonate (SDBS) to fabricate fibril with an average diameter of 35 nm and architecture with a pore size of 160 nm. Such multi-layered fiber structures were useful to attain long-term efficiency for PM_2.5_ and was proven to be better than regular meltblown HEPA filters for their low QF. This architecture could be cleaned for 6 times with ~99.9% efficiency at a pressure drop of ~55 Pa when subjected to 30 L/m of air flow (see Figure 10). However, controlling the nanonet architecture may be bottleneck of this technology. 

#### 4.3.3. Multi-Component and Hybrid Ultrafine PVDF Nanofiber Filter

**Figure 11:**
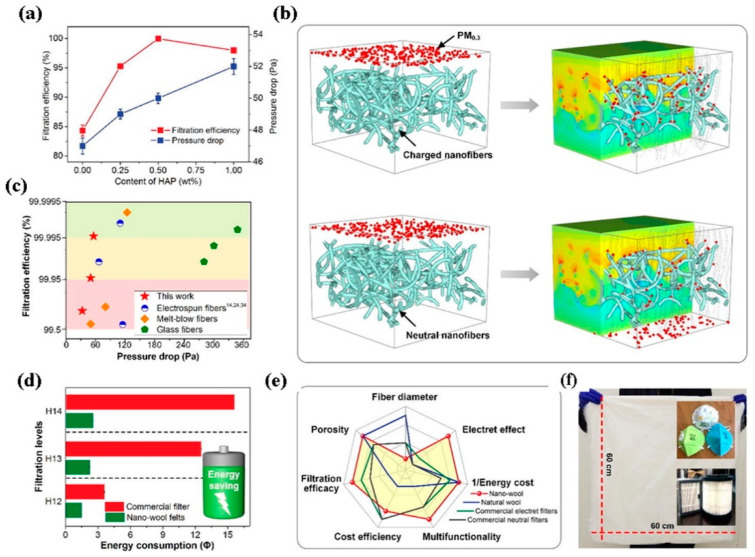
(**a**) Filtration efficiency and pressure drop of PVDF nanowool fibers with varying HAP content, (**b**) filtering efficiency of charged and neutral nanofibers towards the most penetrating particle, (**d**) comparison of filtration efficiency of nanowool fibers with other commercial filters, and (**e**) a radar plot showing a comparison among self-crimp nanofibers, natural wool, and commercial air filtration, and (**f**) design of air filters from PVDF NWF. Reproduced with permission from Ref. [114]. Copyright Elsevier 2020.

In a novel attempt to fabricate a bicomponent nanofibrous architecture a hybrid nanofiber membrane containing side-by-side PVDF/PI fibers was manufactured via electrospinning. In this process, the needles were kept in a side-by-side manner such that Taylor’s cones of two consecutive jets met each other at the outlet of the dual-spinneret configuration [115]. The PVDF/PI fibers had an average diameter of 541 nm and the membrane exhibited high mechanical and thermal stability, making it suitable for high-temperature filtration of particulates. The bicomponent fibers showed PFE of ~96% at 230 °C for PM_0.3_ due to reduced pore size arising from swelling of PVDF. Self-crimped wool such as PVDF nanofibers have been obtained by electrospinning PVDF using DMF as a solvent at various RH such as 30%, 60%, and 90%. The moisture-mediated electrospinning allowed self-curling of nanofibers affected by tailored exchange between DMF and water molecules [116]. The bending perturbations have been found to be much lower at RH 90% producing ultraporous (98.7%) and ultrafine (0.6 μm fibers) wool like architecture that enhances the air flow drastically. The nanofibers were rendered electret using hydroxyapatite (HAP) as charge enhancer, which allowed the nanowool surface potential to be as high as 13.26 kV. Such high electrostatic charge could assist in PM_0.3_ removal at differential pressure of 50 Pa (c.f. Figure 11) [116].

Similar enhancement of electret filter behavior by addition of a second phase particle to PVDF nanofibers was seen elsewhere [110] where authors electrospun PVDF and SiO_2_ (30 nm) nanoparticle mixture modified with γ-glycidoxypropyltrimethoxysilane (GPS) to improve interfacial charged regions of PVDF and SiO_2_. The GPS enhancement led to a significant improvement in total filtration efficiency where 24% contribution came from electret effect apart from mechanical separation, which was otherwise missing in pure PVDF/SiO_2_ composite fibers (performed as complete mechanical separator) [117]. PVDF-trifluoroethylene (PVDF-TrFe) nanofibers were electrospun and deposited on PET mesh to eliminate separate air filtration for HEPA and ULPA, i.e., for particles less than 0.1 μm [118]. Ferroelectric and triboelectric coupling led to an enhancement of the surface electric potential maintaining air permeability and high efficiency. The filtration efficiency was around 88% for polarized nanofibrous net which increased to 94% after triboelectrification and nylon brushing with opposite charging, which is around 99% with PM_2.5_. Triboelectric charging increased identical surface charge for both polarized and non-polarized fibers, in addition to the ferroelectric alignment of the β-form of PVDF fibers, well maintained at a temperature above 110 °C (Curie temperature). The triboelectrically charged PVDF fibers maintained a charge stability of around 50% even after 8 h [118]. Utilization of PVDF-LiNBO_3_ electrospun nanocomposites in window screens as electrets filters was also attempted [119]. Self-polarized 2D nanofibers with a diameter of 21 nm and a pore size of 0.26 μm were constructed, which gave higher MPPS adhesion and interception tendency than nylon 6, PAN, and PU as well as lower pressure drop for PM_0.3_. The mean free path of the air molecules is 3 times higher than the fiber diameter, giving rise to molecular regime flow leading to a quality factor of about 10 times larger than a commercial air filter, as observed for 100 h of testing [119]. This also allowed light transmittance of about 80%, enhancing visibility of window screen as well as a bursting strength of around 175 KPa. However, this filtration efficiency lessens, once the electrospinning melt exceeds the Curie temperature of 125 °C, after which PVDF recrystallizes and membrane porosity decreases. PVDF-Fe_3_O_4_ nanofibrous membrane was developed with glass fiber mesh and PET as backing materials, where the magnetic properties of Fe_3_O_4_ along with the ultrafine nanofiber structure led to a remarkable PFE of ~99.95% for PM_0.3_ at a much lower pressure drop (58.5 Pa), which is far less than a conventional CNT quartz filter. This eventually increases the figure of merit of the nanonet, which is seemingly a useful anti-haze window screen material [120]. The critical concentration of nanoparticles was found to be 1%, after which the filtration efficiency becomes constant.

#### 4.3.4. Biocompatibility of PVDF Nanofiber-Based Filter

The application of nanofibers in facemasks for separation of PMs or dangerous virus containing aerosols are well established, and it is beyond an iota of doubt. However, the long-term effects of nanomaterial incorporation on such PPEs, both in terms of wearer safety and environmental impact after recycling or reuse, are of particular concern [121]. Although the possibility of inhalation of micro-debris of meltblown product-based facemasks (<3 μm sized fibers) leading to cardiac issues, inflammation, respiratory irritation, and stress can pose significant difficulties to wearers and have been studied elsewhere [122], carefully designed sandwiched middle layer of nanofibers may not lead to such deteriorating health effect. PVDF, with fluorine atoms in its backbone, is chemically inert to most of the organic and inorganic compounds. When used in combination with other fibers such as polycaprolactum (PCL), PVDF has been found to be biocompatible in the form of scaffolds as well as nano-generators [123]. However, PVDF has been found to exhibit mild inflammatory tendency when it meets human skin. This effect is considerably less pronounced than that of PP, a common constituent of middle layer in respirators and face masks [124]. To the best of knowledge of authors, there is not enough evidence regarding biotoxicity and potential biohazard of PVDF nanofiber-based air filter, especially facemasks. 

## 5. Conclusions

This review summarizes the progress of ultrafine PVDF nanofiber-based air filters for PM removal in recent years. It is well understood and established that spunbond or meltblown fibers are several microns in diameter, and hence, electrospinning and recently developed solution blowing have emerged as viable processes to develop nanofiber-based filter membrane. Albeit the former of the last two mentioned is more developed, since ~30 companies all around the globe develop fine nanofiber-based filter membrane, especially face mask, the latter is more of an emerging technology, which is more scalable than electrospinning. The beauty of both the processes lies in their capability to produce ultrafine fibers (<500 nm). 

Ultrafine fibers, because of their architecture, allow slip and/or transition flow around their architecture when PM laden air is forced through such fibrous membranes, which is distinctly different than conventional textile or microfiber-based membranes, where continuum flow is expected leading to flow resistance and high drag, also known as increased pressure drop. For face masks, this is commonly identified with reduced breathability. This review systematically explains the beauty of ultrafine fibers and their effect on reduction in pressure drop and increment in PFE via enhanced PM–fiber interaction and enhanced diffusive entrapment probability. PVDF nanofibers, in their ultrafine structure, promote the mechanical separation of PMs from air based on both diffusion and interception. Along with the mechanical separation, PVDF nanofibers can be effectively used as electret filter owing to their innate polarized structure, commonly found in uniaxially stretched PVDF in the β-phase form. In general, electrospinning promotes the formation of β-phase thereby suppressing the commonly found α-phase in PVDF. The former is already well-known piezoelectric material and is used in tactile sensors, actuators, etc. Several researchers have reported the dominance of electret separation mechanism over interception and Brownian diffusion phenomena. The effect of RH, applied electric voltage, solvent ratio, and phase separation while stretching polymer jet, if any, have immense effects on the fabrication of ultrafine fibers and the resultant β-phase structure. While some researchers have fabricated uniform ultrafine fibers and rendered them electret by application of electric field at several kV/cm, some have used the inherent polarized structure, such as nano-net of tree-like nanoweb formation, while tweaking the architecture, which enhances the interaction between PM and fibers, along with enhanced sieving capability. At the same time, triboelectric membranes are also advantageous for PM removal at an efficiency of 99.99%, especially for MPPS. Despite the great number of researches conducted on PVDF nanofibers as an air filtration membrane, there are several technological bottlenecks in this scheme as follows:

- The charge decay and lack of washability of the electret filter membranes, which is also common for general N95 category masks that use an electret meltblown layer in the face masks.
- The charge retention capacity depend on electrical conductivity and localized trap sites. This issue is often tackled with incorporation of the second phase particle, such as SiO_2_, Fe_2_O_3_, etc.
- Charge decay in humid conditions, which can be tackled with superhydrophobic coatings.
- Scalability issues in development of aforementioned nanoweb/tree-like structures, etc.

Despite these issues, the PVDF ultrafine nanofibrous membranes are as feasible as any other polymers. Their excellent chemical inertness, ability to be spun into dimension of ~100 nm using relatively benign solvents, innate polarized structure, and high temperature withstanding capacity make it a very good candidate for development of an excellent PM filter. Researchers even have developed transparent antihaze window screens with such webs, which exhibited excellent PFE; this demonstrates the versatility of the PVDF ultrafine nanofibrous membranes. Although the scope of studying hazardous effects of PVDF nanofiber-based facemasks has remained rather unexplored, it may open up new ventures of research in the said field.

## Data Availability

Data sharing not applicable.
